# Supplementary material for: Cloning and Characterization of Yak DHODH Gene and Its Functional Studies in a Bisphenol S-Induced Ferroptosis Model of Fetal Fibroblasts
Source: Animals (Basel). 2023 Dec 13;13(24):3832. doi: 10.3390/ani13243832 (PMC10740537; doi:10.3390/ani13243832)

Raw data of western blots

(1)
Antibody: DHODH, 14877-1-AP (Proteintech, Wuhan, China);
Calculated molecular weight: 43 kDa;
Sample information (from left to right): the 1-3 bands are the group Ctrl (NC), and the 4-6 bands are the group OE, with three replicates in each group.

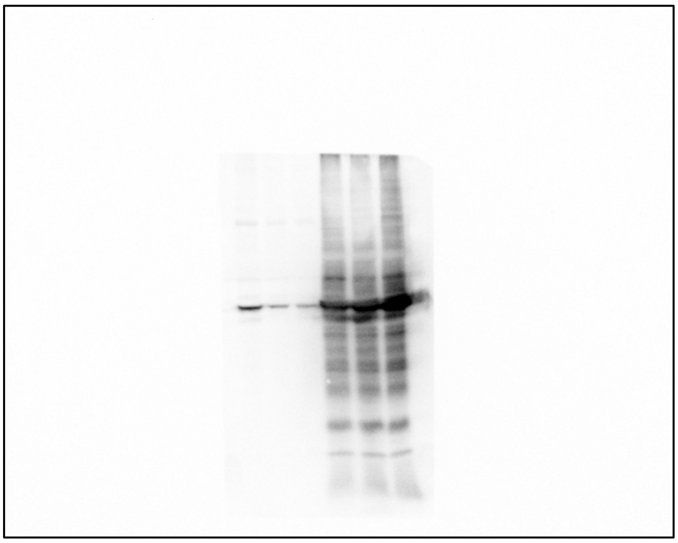

(2)
Antibody: ACTIN, 4967S (Cell Signaling, Danvers, USA);
Calculated molecular weight: 45 kDa;
Sample information (from left to right): the 1-3 bands are the group Ctrl (NC), and the 4-6 bands are the group OE, with three replicates in each group.

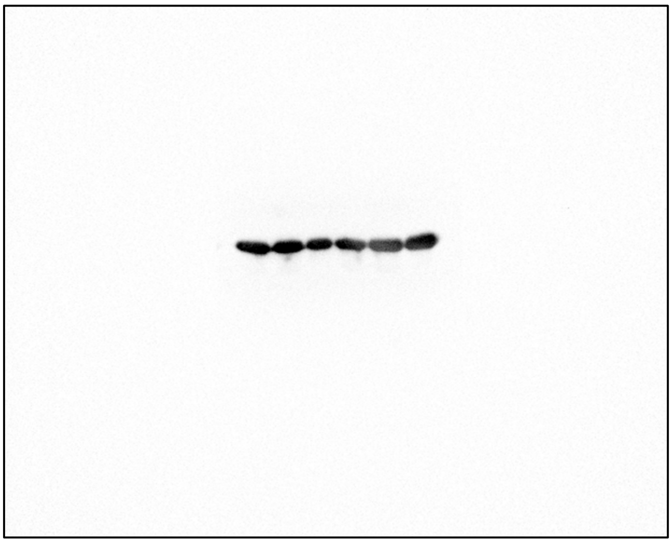

(3)
Antibody: DHODH, 14877-1-AP (Proteintech, Wuhan, China);
Calculated molecular weight: 43 kDa;

Sample information (from left to right): the 1-3 bands are the group Ctrl (NC), and the 4-6 bands are the group SiRNA, with three replicates in each group.

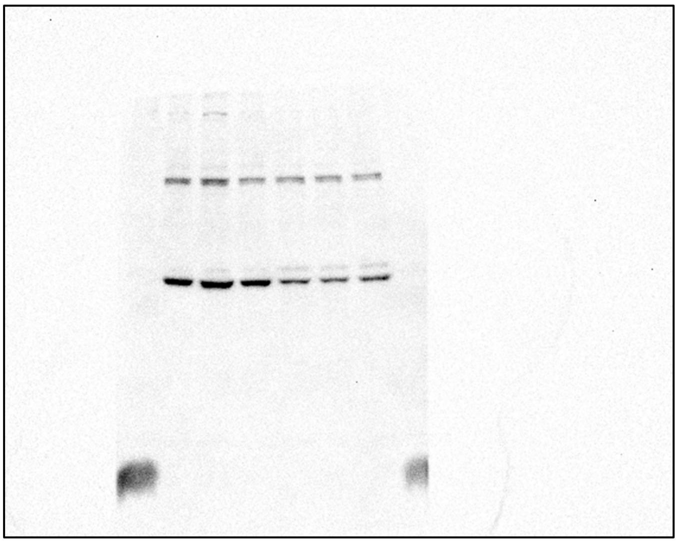

(4)
Antibody: ACTIN, 4967S (Cell Signaling, Danvers, USA);
Calculated molecular weight: 45 kDa;
Sample information (from left to right): the 1-3 bands are the group Ctrl (NC), and the 4-6 bands are the group SiRNA, with three replicates in each group.

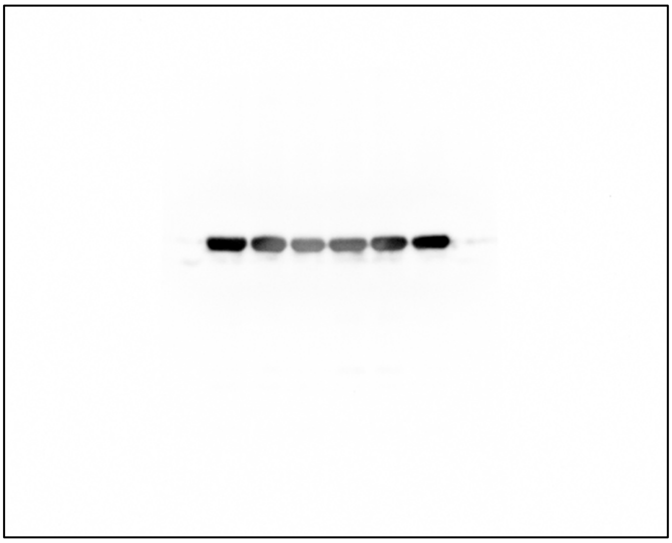

Supplement: Supplementary file 1 [file animals-13-03832-s001.zip › Raw data of western blots for Animals-2694502.pdf]
