# Supplementary material for: Cloning and Characterization of Yak DHODH Gene and Its Functional Studies in a Bisphenol S-Induced Ferroptosis Model of Fetal Fibroblasts
Source: Animals (Basel). 2023 Dec 13;13(24):3832. doi: 10.3390/ani13243832 (PMC10740537; doi:10.3390/ani13243832)
Supplement: Supplementary file 1 [file animals-13-03832-s001.zip › Supplementary Figures.pdf]

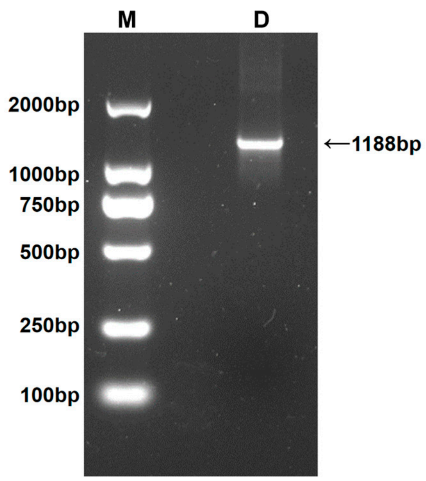

Figure S1. The gel electrophoresis image of PCR products amplified from the coding region of *DHODH* gene. M:DL2000 DNA Marker; D: *DHODH* gene。

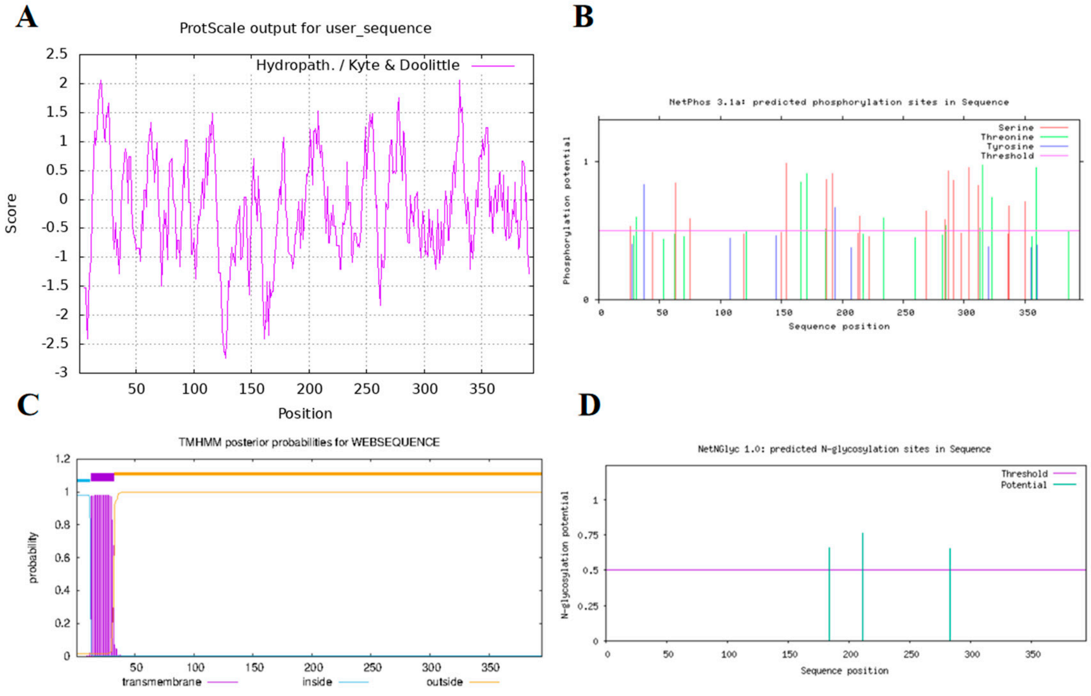

Figure S2. Physicochemical properties analysis of yak predicted DHODH protein. A. Hydropathy property analysis of yak DHODH protein. B. Phosphorylation site analysis of yak DHODH protein. C. Transmembrane structure analysis of yak DHODH protein. D. Glycosylation site analysis of yak DHODH protein.

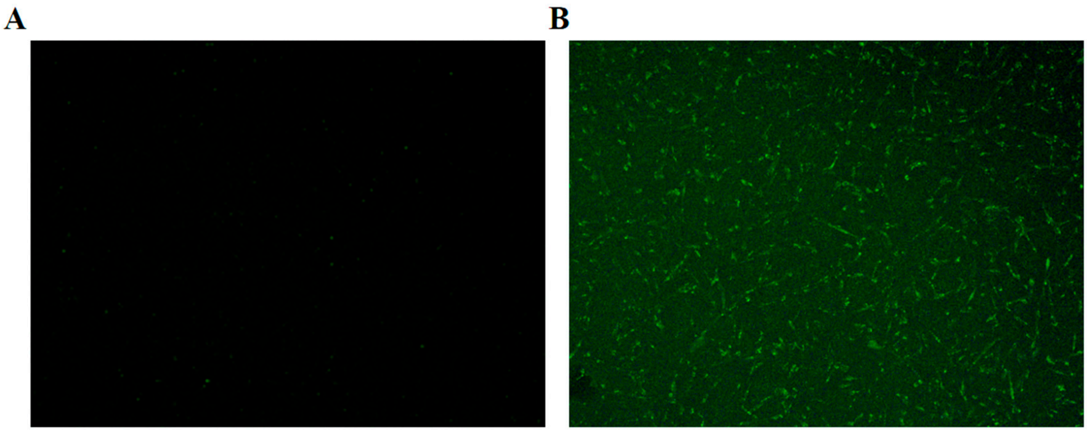

Figure S3. Photographs of YSFs transfecting with negative control vector (A) and *DHODH* overexpressing vector (B). Pictures were captured after vector transfection for 48 h using a fluorescence microscope at the excitation light of 484 nm.
